# Room temperature synthesis of indium tin oxide nanotubes with high precision wall thickness by electroless deposition

**DOI:** 10.3762/bjnano.2.14

**Published:** 2011-02-21

**Authors:** Mario Boehme, Emanuel Ionescu, Ganhua Fu, Wolfgang Ensinger

**Affiliations:** 1 Department of Materials Science, Darmstadt University of Technology, Petersenstr. 23, 64287 Darmstadt, Germany; 2 GSI Helmholtz Centre for Heavy Ion Research GmbH, Planckstraße 1, 64291 Darmstadt

**Keywords:** conductive nanotubes, electroless deposition, indium tin oxide, ion track template, nanotubes

## Abstract

Conductive nanotubes consisting of indium tin oxide (ITO) were fabricated by electroless deposition using ion track etched polycarbonate templates. To produce nanotubes (NTs) with thin walls and small surface roughness, the tubes were generated by a multi-step procedure under aqueous conditions. The approach reported below yields open end nanotubes with well defined outer diameter and wall thickness. In the past, zinc oxide films were mostly preferred and were synthesized using electroless deposition based on aqueous solutions. All these methods previously developed, are not adaptable in the case of ITO nanotubes, even with modifications. In the present work, therefore, we investigated the necessary conditions for the growth of ITO-NTs to achieve a wall thickness of around 10 nm. In addition, the effects of pH and reductive concentrations for the formation of ITO-NTs are also discussed.

## Introduction

Oxide based semiconductive nanostructures have attained a position of significance in science and engineering. For many of these materials reliable syntheses are now available and a wide range of applications in all areas of nanoscience and nanotechnology have become possible [1–3]. In case of oxide based nanomaterials, ITO seems to be very interesting on account of its conductivity and optical properties. Due to its characteristics, ITO nanostructures can be considered as promising materials for the development of applications in optoelectronics, sensors and biomedical sciences [4–6]. Miscellaneous methods for the fabrication of ITO nanostructures, such as the post calcination method [7], alkaline hydrolysis [8] or pulsed laser ablation [9] have been developed and used. For fabricating metal nanostructures, the template deposition method, pioneered by C.R. Martin [10–11], is one of the most important processes. So far, various types of nanostructures obtained by electroless deposition have been successfully fabricated using chemical and physical methods [12–14]. However, the synthesis of ITO nanotubes by electroless deposition has not yet been reported. Electroless deposition has several advantages over the above noted methods. Template based electroless deposition is actually the most cost effective way to generate ITO nanotubes. Moreover, the preparation time of the nanotubes is quite short and the equipment required is available as the standard instrumentation of a chemical laboratory.

In this publication, we describe an effective synthesis of ITO nanotubes (ITO-NTs) with a diameter of approx. 100 nm and a wall thickness of approx. 10 nm. The ITO-NTs were grown by electroless deposition under aqueous conditions in ion track etched polycarbonate templates. Due to surface limitations, electroless deposition is a convenient and power saving method to construct hollow nanostructures. The structural properties of the ITO tubes grown were investigated by various analytical techniques. The growth mechanism of the ITO-NTs was proposed on the basis of the experimental results. Previously zinc oxide films have been prepared by electroless deposition in aqueous solutions under different conditions as shown in Table 1.

**Table 1:** ZnO films prepared in aqueous solution.

Deposition source	Reductant	Temp.	pH	Ref.

ZnCl_2_	CH_4_N_2_O	70 °C	8	[15]
Zn(NO_3_)_2_	C_6_H_12_N_4_	90 °C	n. a.	[16],[17]
Zn(CH_3_COO)_2_	C_2_H_8_N_2_	50 °C	9–12	[18]

Ito et al. obtained highly oriented crystalline films, grown directly on a substrate in aqueous solutions containing urea [19]. Crystalline zinc oxide films were prepared in alkaline aqueous solutions containing ethane-1,2-diamine as a chelating agent [20]. Well aligned ZnO microrods within a thin film were formed by the thermal decomposition of an amino complex based on Zn^2+^ and methenamine [21–22]. All these methods are not adaptable to prepare ITO-NTs, even with modification of the pH, temperature or reductive concentrations. All modifications, based on literature procedures, gave only films consisting of needles, flakes or flower-like structures, although we were able to produce homogeneous layers with thicknesses much greater than the desired outer diameter of the required ITO-NTs.

In the present work, we investigated the essential conditions for the growth of ITO-NTs to achieve a wall thickness below 20 nm, using indium sulfate, tin sulfate and (CH_3_)_2_NH·BH_3_ (dimethylamine borane, DMAB). The effects of pH and reductive concentrations are also discussed in this report.

## Results and Discussion

To begin with we will discuss the reaction mechanism of the three steps prior the ITO deposition step. During the sensitization process, the tin chloride is first hydrolyzed to modify the etched polycarbonate template with Sn^2+^ ions by electrostatic attraction (Equation 1). Subsequently, in an activation step a redox reaction is carried out on the modified template surface by the addition of Ag^+^ ions which oxidizes the Sn^2+^ ions to Sn^4+^ ions. At the same time, Ag^+^ ions are reduced to metallic Ag which form Ag^0^ clusters on the template surface (Equation 2). Using an aqueous PdCl_2_ solution, the Ag^0^ clusters are oxidized to Ag^+^ and pass into solution whilst the Pd^2+^ ions are reduced to Pd^0^ to form palladium clusters on the template surface (Equation 3). Following these steps using the parameter named in the experimental section of this report, we were able to produce more than 2000 clusters per µm², which is one of the keys to achieve ITO nanotubes with the reported dimensions.

Senzitation:

[1]
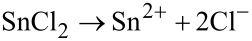


Activation:

[2]
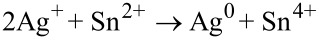


Palladium cluster formation

[3]
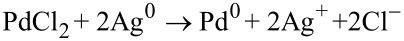


To discuss the growth mechanism of the ITO-NTs induced by electroless deposition method the general chemical deposition mechanism of ITO in the solution can be described as follows:

[4]
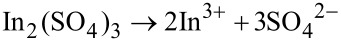


[5]
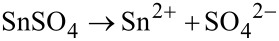


[6]
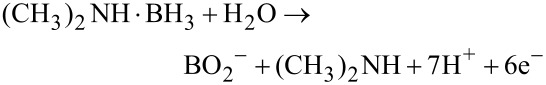


[7]
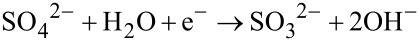


[8]
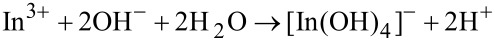


[9]
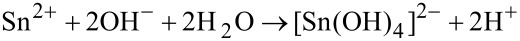


[10]
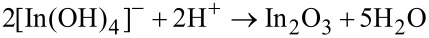


[11]
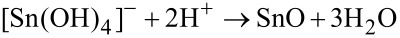


[12]
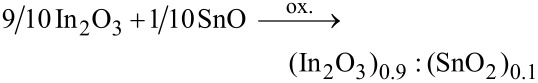


First, In_2_(SO_4_)_3_ and SnSO_4_ are hydrolyzed in the chemical deposition solution (Equation 4 and Equation 5). At the same time, (CH_3_)_2_NH·BH_3_ is also hydrolyzed and free electrons are released (Equation 6) which reduce SO_4_^2−^ ions to SO_3_^2−^ ions and lead to an increase in the OH^−^ concentration (Equation 7). The OH^−^ ions combine with In^3+^ and Sn^2+^ to yield intermediate products such as In(OH)_3_ and Sn(OH)_2_ or [In(OH)_4_]^−^ and [Sn(OH)_4_]^2−^ in solution (Equation 8 and Equation 9) [15]. Because of ionic diffusion, interactions between molecules and ions in the solution and heat convection inside the etched ion tracks, the nanotubes are induced via the reactions shown in Equation 10 and Equation 11. It is well understood that the mode of nucleation, which is heterogeneous nucleation on surfaces of substrates or homogeneous nucleation in solution, is determined by the supersaturated solution [16]. When the solution is supersaturated, nucleation begins; ITO nanocrystallites form on the template surface (Equation 12), according to the concentration ratio of In_2_O_3_ and SnO_2_, due to the palladium clusters placed on the surface prior to deposition. As the reaction proceeds, more ITO crystallites appear in the solution, and the nanotubes are formed. The conditions of the aqueous solutions can be controlled by adjusting the concentrations, temperature, pH and the quantities and type of reducing agent.

Initially, we investigated the best concentration/pH value for ITO deposition at around room temperature. The results of these experiments are summarized in Table 2.

**Table 2:** Diagram of ITO deposition under various conditions at 23 °C.^a^

		pH						
		3	5	6	**7**	8	9	11

(CH_3_)_2_NH·BH_3_ concentration (mol/L)	0.200	−	0	00	00	00	−	−
	0.100	−	0	00	00	00	−	−
	0.075	−	0	0	0	0	−	−
	**0.050**	−	0	**+**	**+**	**+**	−	−
	0.025	−	−	−	−	−	−	−
	0.010	−	−	−	−	−	−	−

^a^**+**: ITO deposition with induced ITO-NTs; 00: strong ITO deposition, no ITO-NTs observed; 0: ITO deposition, no ITO-NTs observed; −: no satisfactory ITO deposition observed.

The best induced ITO-NTs were obtained with 0.05 mol/L (CH_3_)_2_NH·BH_3_ and a pH value of around 7. At pH values of 3 and over 9 we did not observe any precipitation on the template surface at any concentration of the reducing agent. Between pH 5 and 8 we could discern that precipitation occurred with reductant concentrations of between 0.05 and 0.2 mol/L. With concentrations of the reducing agent greater than 0.05 mol/L, we observed deposition of ITO which formed very thick layers or needles and flakes. Only at around pH 7 at a reductant concentration of 0.05 mol/L, were ITO-NTs with a wall thickness close by the desired size obtained.

These previously ascertained deposition conditions were used to obtain further information about the optimum deposition temperature for producing ITO-NTs with a well defined wall thickness. As shown in Table 3, the same reductive ratios as in the previous experiments were used. A constant pH value of 7 was employed to investigate the precipitation behavior at different increments of the reducing agent at various temperatures.

**Table 3:** ITO deposition under various conditions at pH 7.

		Temperature (°C)						
		0	**3**	6	10	20	40	60

(CH_3_)_2_NH·BH_3_ concentration (mol/L)	0.200	0	00	00	00	00	00	00
	0.100	0	0	00	00	00	00	00
	0.075	−	**+**	**+**	0	0	0	0
	**0.050**	−	**++**	**+**	**+**	**+**	0	00
	0.025	−	−	−	−	−	−/0	0
	0.010	−	−	−	−	−	−	−/0

^a^**++**: best achieved ITO deposition with well defined ITO-NTs; +: ITO deposition with induced ITO-NTs; 00: strong ITO deposition, no ITO-NTs observed; 0: ITO deposition, no ITO-NTs observed; −: no satisfactory ITO deposition observed.

Table 3 shows the change of precipitation behavior. Deposition occurs as thick films or needles and flakes when the temperature of the chemical deposition bath is over 10 °C at a reductant concentration of over 0.05 mol/L. Below 3 °C, deposition takes place only at reductant concentrations over 0.1 mol/L and produced no ITO-NTs. With concentrations of the reducing agent below 0.05 mol/L, precipitation was observed and only at a higher temperature and gave poor quality ITO-NTs. The best result for ITO-NTs having thin walls was achieved at a temperature of 3 °C with a reductant concentration of 0.05 mol/L at pH 7.

**Figure 1:**
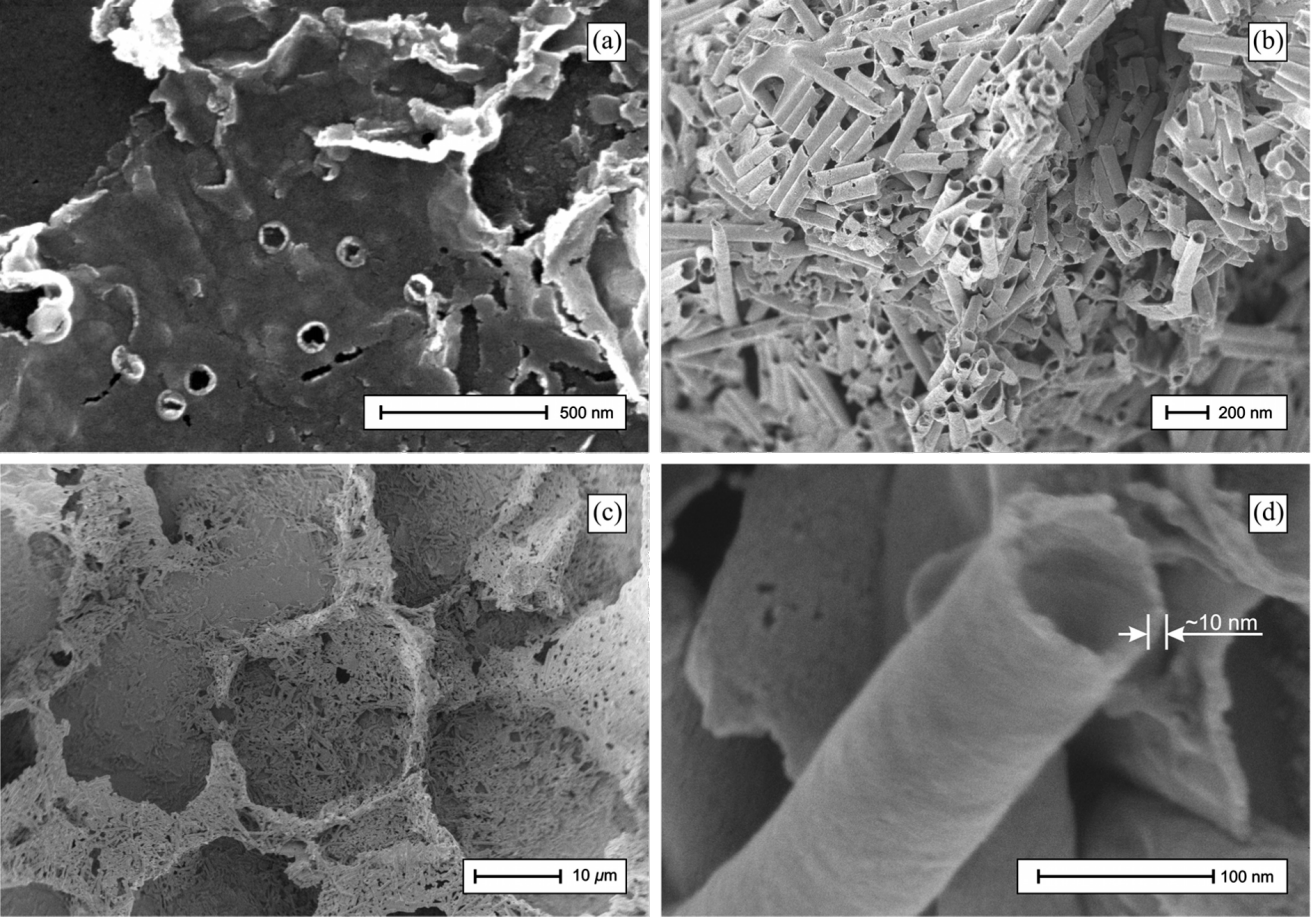
(a) SEM image of the removed ITO layer from the template surface. (b)–(d) SEM images of bulk ITO-NTs freed from the polycarbonate template.

By using polycarbonate templates for electroless deposition, not only does deposition occur in the inner walls but also on the template surface. To use the template as feedstock for ITO-NT arrays, a minimum of one side of the coated surface must be removed. To achieve bulk ITO-NTs both sides of the coated template have to be removed before dissolving the template. We found that the best way to remove the coated surface without damaging the fabricated tubes is to use adhesive tape. Figure 1a shows the surface of the adhesive tape – previously the adhesive side – with the removed template surface deposit sticking to the adhesive. As shown in Figure 1a the removed ITO layer from the template surface mostly contains round “nicks” following breaking up the nanotube/surface assembly and leaving the nanotubes in the template. In this work, both sides were removed before dissolution of the polycarbonate for further investigation.

### Characterization

After the plating process, scanning electron microscopy (SEM) was used for morphology and size distribution investigations of the nanostructures. The polycarbonate covering the ITO-NTs was easily removed with dichloromethane. Energy dispersive X-ray (EDX) analysis was performed to determine the elemental composition. The chemical composition of the ITO-NTs was analyzed by X-ray photoelectron spectroscopy (XPS) using monochromatic Al Kα radiation (*h*ν = 1486.6 eV). In addition to the XPS analysis, photoluminescence was used to prove that the nanotubes consisted of ITO. The ITO-NTs fluorescence emission at room temperature was observed with a fluorescence spectrophotometer (Cary Eclipse, Varian) at an excitation wavelength of 431 nm. Additionally, to obtain more details of the composition of the nanotubes, the nanotubes were examined by Raman spectroscopy with 633 nm laser excitation.

The SEM images in Figure 1b–c show the ITO-NTs freed from the polycarbonate template. The outer diameter of the tube shown in Figure 1d is about 100 nm, which is in relation to the etched ion-track pore size of the polycarbonate. Using high resolution SEM, the surface of the ITO-NTs is observed to be even and clogged under high magnification.

As expected, the nanotubes have open ends, indicating that the deposition of ITO solely occurred on the etched ion track walls. The wall thickness of the tubes is closely related to the electroless deposition conditions. Using the introduced deposition method, it is possible to design an exact desired wall thickness simply by adjusting the deposition time. With short deposition time tubes were achieved, while a prolonged deposition time leads to increased the wall thickness and nanowires with a small open core along their longitudinal axis. The reproducibility of the deposition method using polycarbonate templates was investigated by examination of a significant number of tubes obtained at different deposition times.

**Figure 2:**
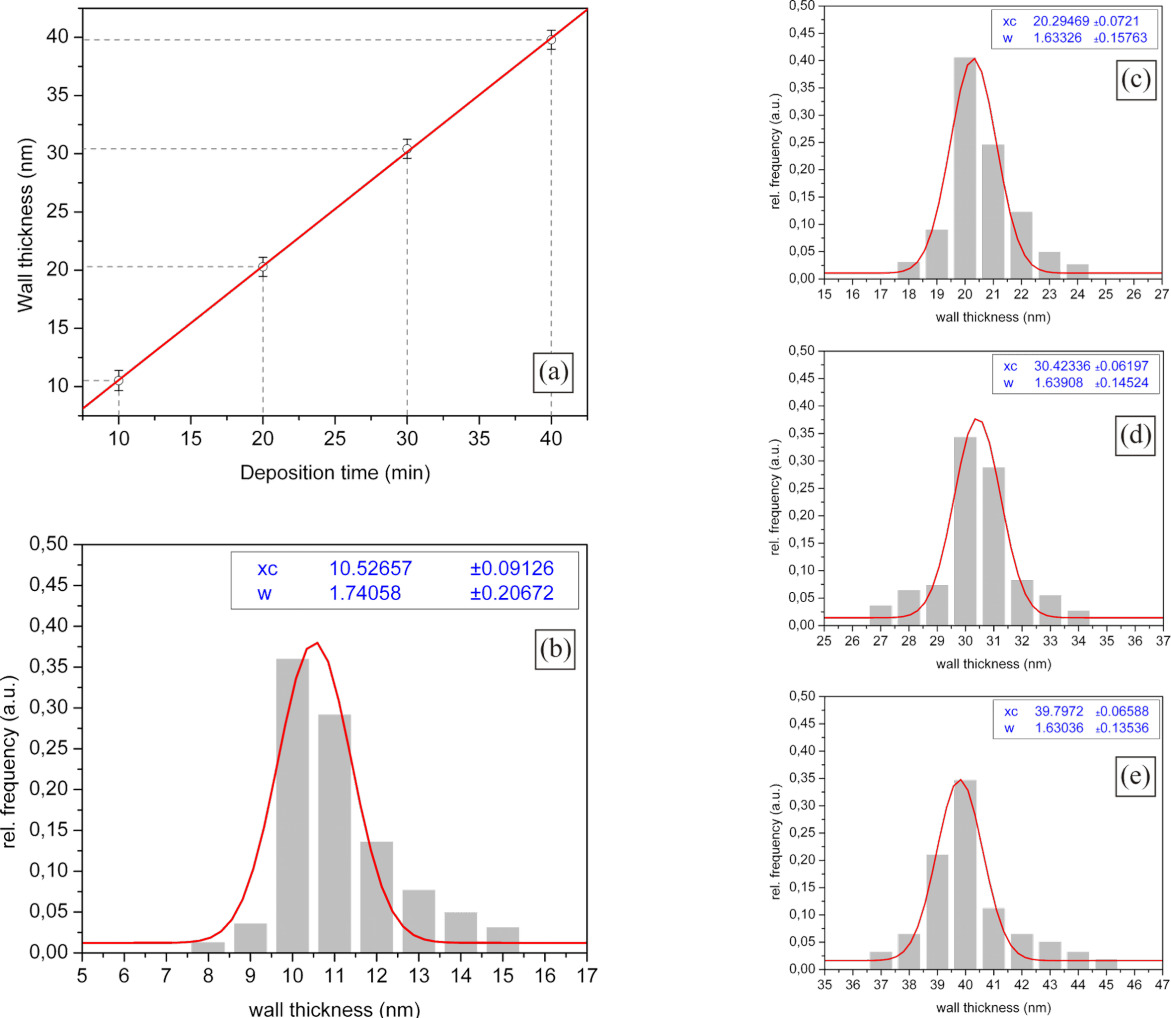
(a) Deposition time vs. wall thickness plot. (b) Gaussian distribution of 10 nm wall thickness ITO-NTs. (c–e) Gaussian distribution of 20, 30 and 40 nm wall thickness ITO-NTs.

To investigate the repeatability of the described method, four different samples with ITO-NTs with an expected wall thicknesses of 10, 20, 30 and 40 nm were prepared. Each sample was prepared so that a bulk material sample was available for SEM investigation, similar to the sample shown in Figure 1b and Figure 1c. The samples were manually scanned for tubes having a position their wall thickness could be measured. Around 150–200 tubes of each sample were surveyed to get best results for a Gaussian distribution wall thickness plot as shown in Figure 2b–d.

For each bar plot shown in Figure 2b–d, a computer aided Gaussian plot was added using the following equation,

[13]
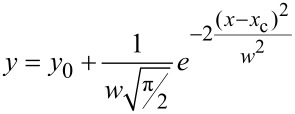


to get further information about the wall thickness (*x*_c_) and the average deviation of the nanotubes. The maximum of the Gaussian distribution (*x*_c_) of each expected wall thickness is equal to the average produced wall thickness of the particular sample and was plotted (Figure 2a). The error bars used in Figure 2a are the result of the 2 sigma values *w* of the Gaussian distribution of the respective sample. Moreover, the diagram in Figure 2a describes the interdependence of deposition time and resulting wall thickness. During the research, it transpired that the deposition rate could be varied using the DMAB concentration and the temperature. Thus, we designed the method to achieve a deposition rate approximately on the scale of 1 nm per minute.

As a result Figure 1 shows, using the deposition method previously described in this report, it is possible to produce ITO-NTs with defined wall thickness of high accuracy. In numbers this means that, if the desired wall thickness is 10 nm, the maximum of the Gaussian distribution for the effective produced wall thickness is 10.53 nm having an irregularity of ± 0.9 nm.

**Figure 3:**
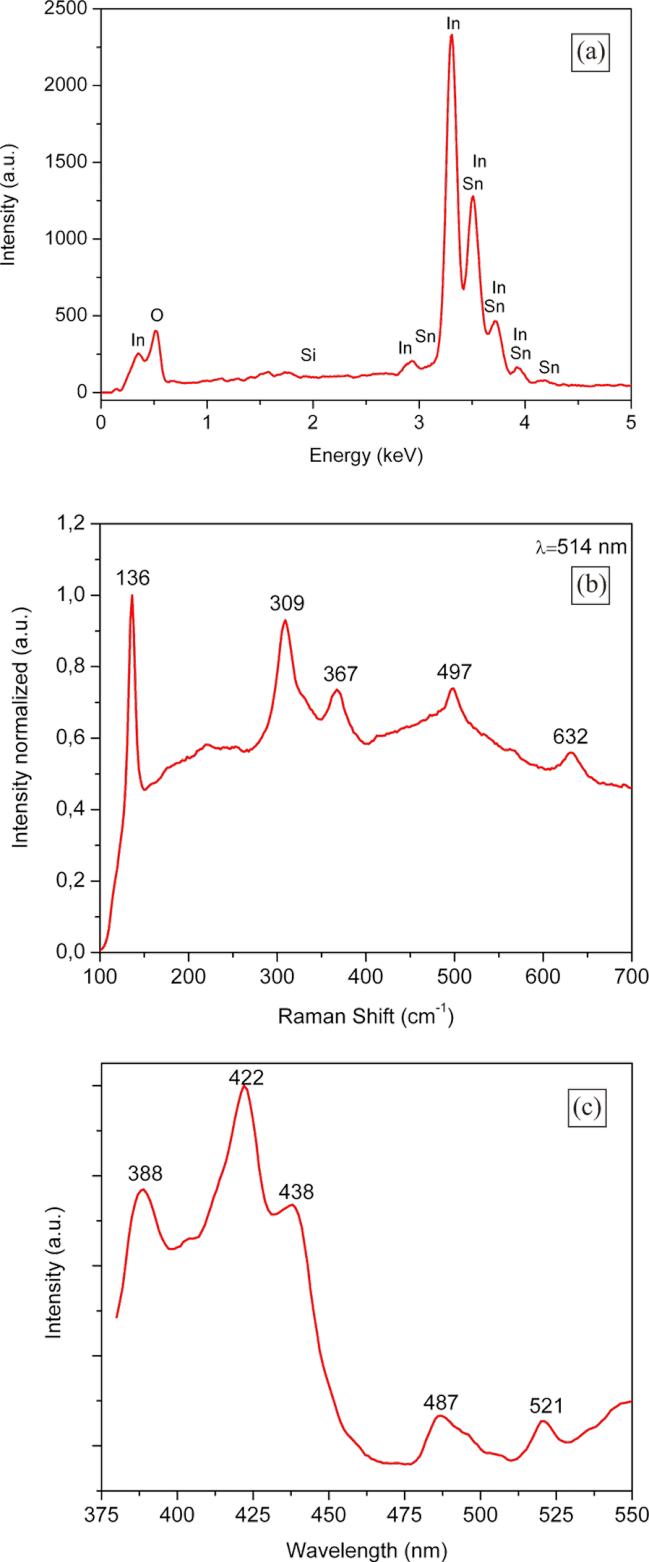
(a) EDX spectrum of ITO-NTs dissected on silicon waver. (b) Normalized Raman Scattering spectrum of the ITO-NTs. (c) Normalized PL spectra of the ITO-NTs excited at 270 nm.

The EDX spectra of the freed ITO-NTs shown in Figure 3a feature peaks corresponding to the elements, In, Sn and O, which correspond to the characteristic composition of the desired ITO-NTs and confirms the presence of ITO. The Si peak arises from the silicon wafer the tubes were stored on for handling purposes. The Raman spectrum for the grown ITO-NTs is shown in Figure 3b.

In the present study, the excitation wavelength is 514 nm. The laser power on the sample surface is about 8 mW and the spot size is 1.5 µm in diameter. The frequency and symmetry of the fundamental Raman active phonon modes 136 cm^−1^, 309 cm^−1^, 367 cm^−1^, 497 cm^−1^ and 632 cm^−1^ for the fabricated ITO-NTs are similar to the vibrational modes previously reported for ITO [23–25].

Figure 3c shows the fluorescence behavior of the fabricated ITO-NTs. The excitation wavelength used in the study was 270 nm. The fluorescence spectrum shows strong UV emission peaks at 388 nm, 422 nm, 438 nm, 487 nm and 521 nm as reported for ITO nanostructures [26–27] and confirms one more the presence of ITO.

**Figure 4:**
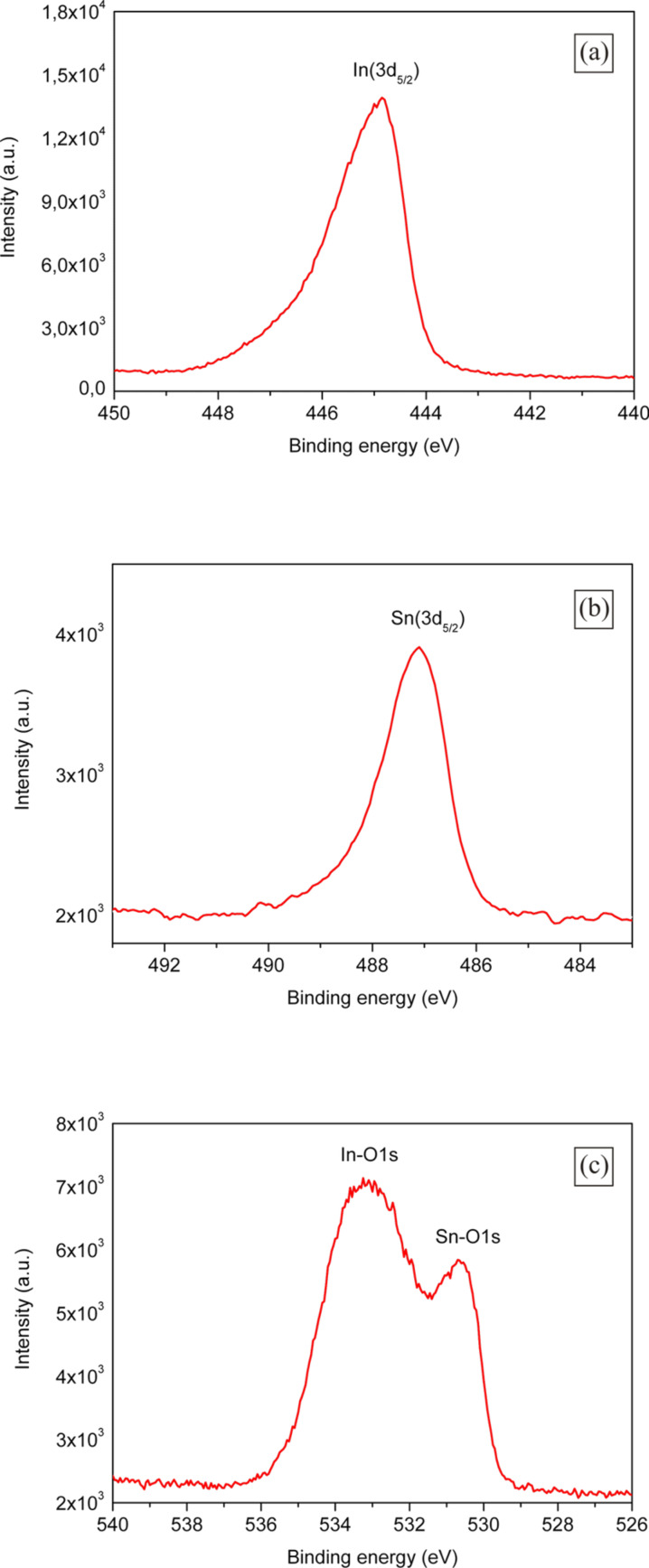
XPS spectra of the synthesized ITO-NTs (a) In(3d) spectrum; (b) Sn(3d) spectrum; (c) In-O(1s) and Sn-O(1s) spectrum.

The composition of the fabricated ITO-NTs was further determined using XPS. The resulting X-ray photoelectron spectra is shown in Figure 4a–c. Figure 4a shows the XPS spectrum in the In(3d) region for ITO, which is located at 445.1 eV. The peak of the XPS spectrum shown in Figure 4b was observed at 487.0 eV is related to the Sn(3d) binding energy. Both peaks illustrated in Figure 4c are focused on the oxygen species in the O(1s)-region. One of the peak maxima is at 530.8 eV and corresponds to Sn-O(1s) whilst the second peak with its maximum at 533,0 eV is for the In-O(1s). Comparing the binding energies shown in Figure 4a–c with literature values [28–29], the composition of the fabricated nanotubes was consistent with the stoichiometry of the ITO.

## Conclusion

Bilateral open cylindrical ITO-NTs with controllable diameter and wall thickness were fabricated at room temperature by electroless deposition in aqueous solutions. This method can be extended to other materials. The developed ITO-NTs have many potential applications. Using polycarbonate as a template, it is possible to produce ITO-NT arrays within the size of the polycarbonate template having a tube density from 1 to 10^9^ tubes/cm². These arrays can be used directly for sensor, photovoltaic or electronic applications.

## Experimental

### Sample preparation

Polycarbonate foils with thicknesses of 6 to 30 µm were used as templates. Commercial polycarbonate (PC) membrane filters, exposed in a very controlled way to charged particles in a nuclear reactor, were obtained from Whatman/GE™ and comparable companies. Because the surface of the commercial membrane filters was treated with polyvinylpyrrolidone (PVP), an additional etching process was used to remove the PVP. Thus, polycarbonate foils were chemically etched at 50 °C with 6 N NaOH solution containing 1% sodium dodecylsulfate [30]. The resulting pore diameter increases linearly with etching time; the pores are cylindrical in shape. In this report, we produced templates with a pore diameter of approx. 100 nm.

### Sensitization and activation

Prior to the electroless deposition process, the surface of the polycarbonate template was treated with sensitization and activation solutions. In this way, the template surface becomes catalytically activated and deposition on the surface is possible. The best results were obtained by the following procedure. The sensitization was performed by an aqueous SnCl_2_ solution containing 0.25 mol/L SnCl_2_ and 0.3 mol/L hydrochloric acid for approx. 30 minutes at 45 °C. After rinsing the sensitized template with deionized water for one minute, it was placed in the aqueous activation solution containing 0.2 mol/L AgNO_3_ and 0.02 mol/L Co(NO_3_)_2_ for a minimum of 10 minutes, followed by rinsing in deionized water for one minute. To complete the activation process the template was placed for a minimum of 15 minutes in an aqueous solution containing 0.2 mol/L Pd(NO_3_)_2_, 0.015 mol/L Ag_2_(SO)_4_, 0.15 mol/L hydrochloric acid and 0.1–0.3 mL of tetrafluoroboric acid at 23 °C. After rinsing with deionized water for 1 minute, the template was prepared for electroless deposition.

### Electroless deposition

The last step is the electroless deposition of ITO. To obtain ITO nanostructures preferably ITO-NTs, the sensitized and activated template was dipped into an aqueous solution containing 0.27 mol/L In(SO_4_)_3_, 0.03 mol/L SnSO_4_ and 0.05 mol/L (CH_3_)_2_NH·BH_3_. The deionized water used was oxygenated in advance to give an oxygen concentration of around 13 mg/L. For the best results, the temperature of the deposition bath was 3 °C. At the end of the deposition time, the template with the inner grown ITO-NTs was rinsed with deionized water for several minutes. The time of deposition can be varied to obtain the desired wall thickness.

## References

[1] Prinz G A (1998). Science.

[2] Kappler J, Tomescu A, Barsan N, Weimar U (2001). Thin Solid Films.

[3] Baughman R H, Zakhidov A A, de Heer W A (2002). Science.

[4] Dhand C, Singh S P, Arya S K, Datta M, Malhotra B D (2007). Anal Chim Acta.

[5] Xu S, Shi Y (2009). Sens Actuators, B.

[6] Shrestha S, Yeung C M Y, Nunnerley C, Tsang S C (2007). Sens Actuators, A.

[7] Chen S-G, Li C-H, Xiong W-H, Liu L-M, Wang H (2005). Mater Lett.

[8] Pramanik N C, Biswas P K (2002). Bull Mater Sci.

[9] Murali A, Barve A, Leppert V J, Risbud S H, Kennedy I M, Lee H W H (2001). Nano Lett.

[10] Martin C R (1994). Science.

[11] Klein J D, Herrick R D, Palmer D, Sailor M J, Brumlik C J, Martin C R (1993). Chem Mater.

[12] Valenzuela K, Raghavan S, Deymier P A, Hoying J (2008). J Nanosci Nanotechnol.

[13] Shi Z, Wu S, Szpunar J A (2006). Nanotechnology.

[14] Porter L A, Choi H C, Ribbe A E, Buriak J M (2002). Nano Lett.

[15] Li W J, Shi E W, Zhong W Z, Yin Z (1999). J Cryst Growth.

[16] Izaki M, Omi T (1997). J Electrochem Soc.

[17] Izaki M, Katayama J (2000). J Electrochem Soc.

[18] Mullin J W (1993). Crystallization.

[19] Ito K, Nakamura K (1996). Thin Solid Films.

[20] O'Brien P, Saeed T, Knowles J (1996). J Mater Chem.

[21] Vayssieres L, Keis K, Lindquist S, Hagfeldt A (2001). J Phys Chem B.

[22] Vayssieres L, Keis K, Lindquist S, Hagfeldt A (2001). Chem Mater.

[23] Berengue O M, Rodrigues A D, Dalmaschio C J, Lanfredi A J C, Leite E R, Chiquito A J (2010). J Phys D: Appl Phys.

[24] Wu K R, Ting C-H, Liu W-C, Lin C-H, Wu J-K (2006). Thin Solid Films.

[25] Shigesato Y, Hayashi Y, Masui A, Haranou T (1991). Jpn J Appl Phys.

[26] Kundu S, Biswas P K (2005). Chem Phys Lett.

[27] Kundu S, Biswas P K (2006). Chem Phys Lett.

[28] Ishida T, Kobayashi H, Nakato Y (1993). J Appl Phys.

[29] Kim J S, Ho P K H, Thomas D S, Friend R H, Cacialli F, Bao G-W, Li S F Y (1999). Chem Phys Lett.

[30] Chtanko N, Molares M E T, Cornelius T, Dobrev D, Neumann R (2004). J Phys Chem B.

